# In Vitro Cytotoxicity of Fluorinated Quaternary Ammonium Salts in Colorectal Cancer Cells and In Silico Pharmacology

**DOI:** 10.1155/2024/2671547

**Published:** 2024-10-30

**Authors:** Adriana Milena Olarte Aponte, Victoria Ospina, Sergio A. Pulido, Luz Amalia Ríos-Vásquez, Luz Adriana Betancur Jaramillo, Carlos Mario Muñetón Peña, Rogelio Ocampo-Cardona, Sara M. Robledo

**Affiliations:** ^1^PECET-Facultad de Medicina, Universidad de Antioquia, Medellín, Colombia; ^2^Grupo Estudios Preclínicos, Corporación de Innovación para el Desarrollo de Productos, Medellín, Colombia; ^3^División I+D+i, LifeFactors Zona Franca S.A.S, Rionegro, Colombia; ^4^Química Teórica y Bioinformática, Departamento de Química, Universidad de Caldas, Manizales, Colombia; ^5^Genética Médica, Facultad de Medicina, Universidad de Antioquia, Medellín, Colombia

**Keywords:** biopharmaceutical properties, colon rectal cancer cell lines, epigenetic analysis, genetic background, physicochemical properties

## Abstract

Colorectal cancer (CRC) is a multifactorial disease driven by genetic and epigenetic alterations that modulate specific metabolic pathways. Despite the availability of effective treatments like 5-fluorouracil (5-FU), pharmacological therapy for CRC still faces significant challenges, including drug resistance, toxicity, and limited specificity. Therefore, discovering new compounds remains critical to overcoming these barriers and expanding treatment options. This study evaluated the cytotoxicity of fluorinated quaternary ammonium salts (FQAS) library in CRC-derived cell lines with premetastatic and metastatic phenotypes. The genetic and epigenetic background of the CRC cell lines and the selectivity of cytotoxicity compared to nontumor cells and between different CRC stages were also assessed. Additionally, the in silico pharmacological properties of these FQASs were analyzed. Results showed that FQASs **9–14** exhibited significant cytotoxic activity against both premetastatic and metastatic CRC cell lines, with FQASs **9**, **13**, and **14** displaying selective toxicity toward CRC cells over normal murine colorectal cells. However, in silico studies indicated poor oral bioavailability for these compounds, suggesting that an injection-based delivery route may be more effective for targeting CRC cells. In conclusion, CF_3_-containing FQASs are promising therapeutic candidates for CRC treatment.

## 1. Introduction

Colorectal cancer (CRC) is a multifactorial and heterogeneous disease that involves genetic and epigenetic alterations that modulate specific metabolic pathways [1, 2]. This neoplasm is the third most prevalent type of cancer and the second leading cause of cancer-related deaths [3]. In Colombia, CRC ranks fourth in prevalence and incidence and third in mortality rates [3], constituting a public health problem. Factors contributing to the development of CRC include age, family history, chemical exposure, western diet, smoking, mutations in tumor suppressor genes and oncogenes, microsatellites alterations, chromosomal instability (CIN), and epigenetic background [4, 5].

Oncogenic transformation in CRC is primarily driven by gene mutations such *as APC*, *KRAS*, *SMAD4*, and *TP53*, collectively known as the adenoma–carcinoma sequence (ACS) [6, 7]. Additionally, epigenetic changes, particularly DNA methylation and histone modifications, play crucial pathophysiological roles in CRC initiation and progression [6, 8]. On the other hand, hypermethylation of CpG islands in promoter regions of tumor-suppressor genes placed by DNA methyltransferases (DNMTs) inhibits gene expression, favoring tumorigenesis, while hypomethylation of promoters or distant super-enhancers can enhance the expression of proto-oncogenes [7–9].

CRC management relies on various systemic interventions, including resection surgery, chemotherapy, biotherapy, and immunotherapy [10–12]. Despite the availability of effective treatments like 5-fluorouracil (5-FU), a drug widely used in the pharmacological therapy for CRC, still faces significant challenges, including drug resistance, toxicity, limited specificity, and high cost [11–13]; in addition, genetic background is also a determinant factor for the therapeutic outcomes [11, 13]. Therefore, discovering new compounds remains critical to overcoming these barriers and expanding treatment options.

Quaternary ammonium salts (QASs) are a large family of organic salts characterized by at least one quaternary ammonium cation, where a nitrogen atom is covalently bonded to four carbon atoms [14]. These compounds have demonstrated a range of biological activities, including antiviral [15, 16], antibacterial [17], antifungal [18], and antitumoral effects [14, 19, 20]. Additionally, some QASs feature fluorine atoms or trifluoromethyl groups attached to the phenyl rings of their vinyl moieties, which have been associated with leishmanicidal [21] and anti-trypanosomal activities [22].

Fluorinated moieties, in general, are widely used in medicinal compounds due to the unique properties fluorine atoms confer. With a size like hydrogen but significantly higher electronegativity, fluorine's nonpaired electrons and strong C–F bonds often enhance the physicochemical properties of molecules, potentially improving biological activity or slowing metabolic degradation [23–25]. Trifluoromethyl groups (–CF_3_) can alter important drug characteristics such as steric effects, lipophilicity, pKa, and the capacity to form hydrogen bonds, all of which can significantly influence pharmacokinetic parameters like absorption and metabolism [26]. Similarly, recent studies suggest that the biological activity of QASs is shaped not only by their chemical structure but also by their hydrophilic and hydrophobic properties, pointing to specific biological mechanisms underlying their cytotoxic effects [27–29]. Therefore, by highlighting the influence of both fluorinated groups and the structural properties of QASs, these findings underscore the potential for designing more effective and targeted pharmacological agents.

The present work aims to design and synthesize a series of novel fluorinated QASs (FQASs) of general structure {[(*Y*-C_6_H_5_)_2_C=CH-(CH_2_)_n_]N(CH_3_)_2_CH_2_*X*}^+^ I^−^ with different lengths in the carbon chain and variations in the fluorinated position, either *meta* or *para* positions and the subsequent determination of the cytotoxicity effects of each compound over three CRC-derived cell lines with different metastatic phenotypes, according to its genetic and epigenetic molecular phenotypes. The potential pharmacology of fluorinated and nonfluorinated QASs was also assessed using in silico approaches to identify promising hit molecules for developing specific antitumoral therapies for CRC.

## 2. Results and Discussion

### 2.1. Synthesis and Characterization of FQASs and QASs

**Figure 1:**
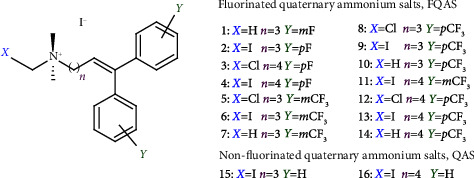
Generic structure of fluorinated and nonfluorinated quaternary ammonium salts.

The 14 FQASs synthesized were: C_5_CH_3_mF_2_ (**1**), C_5_IpF_2_ (**2**), C_6_ClpF_2_ (**3**), C_6_IpF_2_ (**4**), C_5_ClmCF_3_ (**5**), C_5_ImCF_3_ (**6**), C_5_CH_3_mCF_3_ (**7**), C_5_ClpCF_3_ (**8**), C_5_IpCF_3_ (**9**), C_5_CH_3_pCF_3_ (**10**), C_6_ImCF_3_ (**11**) C_6_ClpCF_3_ (**12**), C_6_IpCF_3_ (**13**), and C_6_CH_3_pCF_3_ (**14**). The two synthesized QASs corresponded to C_5_I (**15**) and C_6_I (**16**) (Figure 1).

### 2.2. Genetic and Epigenetic Analysis of CRC Cell Features

Understanding the genetic and epigenetic landscape of CRC cell lines with premetastatic, metastatic in situ, and metastatic invasive phenotypes is essential for identifying novel therapeutic targets, predicting drug efficacy, and developing more effective treatments for CRC. In the CRC cell lines studied (SW948, SW48, and SW620), eight mutated genes were identified (Table 1), all of which play crucial roles in gene regulation, cell proliferation, repair, angiogenesis, and metastasis. Any mutation in these genes may disrupt normal cell cycle control and proliferation processes.

The *TP53* gene was mutated in all three cell lines (premetastatic SW948, metastatic in situ SW48, and metastatic invasive SW620), underscoring their fundamental role in cell cycle control, senescence, apoptosis, and DNA repair. The common R273H mutation in *TP53* was observed across these lines, a mutation known to maintain the regular conformation of the TP53 protein while inducing apoptosis [7]. However, this mutation also contributes to cell cycle dysregulation, affecting normal proliferation and compromising DNA repair mechanisms [31, 32].

The *KRAS*, *PIK3CA*, and APC genes were mutated in both premetastatic SW948 and metastatic invasive SW620 cell lines. These genes are involved in pathways that regulate the cell cycle, proliferation, and angiogenesis [9]. Specifically, mutations in the *APC* gene are linked to enhanced cell survival by modulating proteins such as BCL2, IAP, PCNA, eNOS, and A20, promoting abnormal cell proliferation [33, 34].

Uniquely, SW620 cells (metastatic invasive) also exhibited mutations in the *SMAD4* gene, which is associated with promoting metastasis [35]. This finding suggests that the *SMAD4* mutation could play a critical role in the progression from localized to invasive metastatic phenotypes. In contrast, the metastatic in situ SW48 cells showed distinct genetic mutations. Alongside *TP53*, SW48 cells presented mutations in *CTNNB1*, *EGFR*, and *FBXW7*, genes known for their involvement in oncogenesis and tumor progression [30, 36, 37]. These mutations could influence cell signaling pathways that drive tumor growth and potentially contribute to the early stages of metastasis.

Overall, the differences in molecular phenotypes and allelic expression of mutated genes between these CRC cell lines reflect the varying stages of metastatic potential. Mutations in *TP53*, *KRAS*, *PIK3CA*, and *APC* are shared by premetastatic and metastatic invasive cells, while *SMAD4* mutations are specific to invasive cells, suggesting its role in metastasis. The distinct mutation profile of SW48 highlights the importance of *CTNNB1*, *EGFR*, and *FBXW7* in metastatic in situ phenotypes.

On the other hand, understanding the molecular characteristics of CRC cell lines, including their microsatellite instability (MSI), microsatellite stability (MSS), CIN, and CpG island methylator phenotype (CIMP) status, is crucial for studying not only their biology and disease progression but also therapeutic susceptibility. Our analysis showed that SW48 cells are MSI-positive, while SW620 and SW948 are MSS (Table 2).

The MSI-positive status in SW48 has important biological implications, influencing processes such as tumor development and progression, response to therapy, molecular subtyping, and genomic stability. MSI is typically associated with defects in the DNA mismatch repair system, leading to the accumulation of mutations in microsatellite regions, which contributes to cancer initiation and progression. As a result, SW48 cells, with their MSI-positive status, may exhibit enhanced tumor development and progression compared to MSS cells like SW620 and SW948. Furthermore, MSI-positive tumors, such as those derived from SW48 cells, tend to be more responsive to immunotherapy, particularly immune checkpoint inhibitors, making this cell line a valuable model for exploring therapeutic responses.

The increased genomic instability seen in MSI-positive cells like SW48 results from the accumulation of mutations, affecting crucial cellular processes such as proliferation, differentiation, and survival. In contrast, SW620 and SW948, being MSS, exhibit a more stable genomic environment. The MSS phenotype may correlate with lower mutation rates in microsatellite regions, and consequently, these cell lines might demonstrate different responses to therapeutic agents, particularly those targeting DNA repair pathways.

Regarding the CIMP profile, both SW48 and SW620 cells are CIMP-positive (Panels 1 and 2), indicating the presence of aberrant DNA methylation patterns commonly associated with altered gene expression and tumor behavior. CIMP-positive status can influence epigenetic regulation of oncogenes and tumor suppressor genes, contributing to tumor aggressiveness and resistance to some therapies. The presence of aberrant methylation in these cell lines suggests they may have distinct epigenetic landscapes, making them valuable models for exploring therapies targeting DNA methylation pathways.

On the other hand, SW948 is CIMP-negative but CIN-positive, reflecting increased chromosomal aberrations and aneuploidy, which contribute to tumor heterogeneity and progression. CIN-positive tumors are often characterized by high CIN, which may lead to resistance to certain treatments but could also provide opportunities for targeted therapies that exploit this instability. Notably, SW48 and SW620 cells are CIN-negative, signifying a more chromosomally stable profile. The lack of CIN in these cell lines might suggest that their tumorigenic potential is more strongly driven by epigenetic changes (CIMP) and mutations in key oncogenes or tumor suppressor genes rather than CIN.

### 2.3. Cytotoxicity of FQASs and QASs

The cytotoxicity of FQASs (compounds **1**–**14**) and QASs (compounds **15**-**16**) was evaluated in CRC cell lines SW48, SW620, and SW948, along with healthy murine colorectal tissue (MCRT) and peripheral blood mononuclear cells (PBMCs), using the half-maximal cytotoxic concentration (CC_50_) values (*μ*M) and the cytopathic effect (CPE). Results are summarized in Table 3.

**Figure 2:**
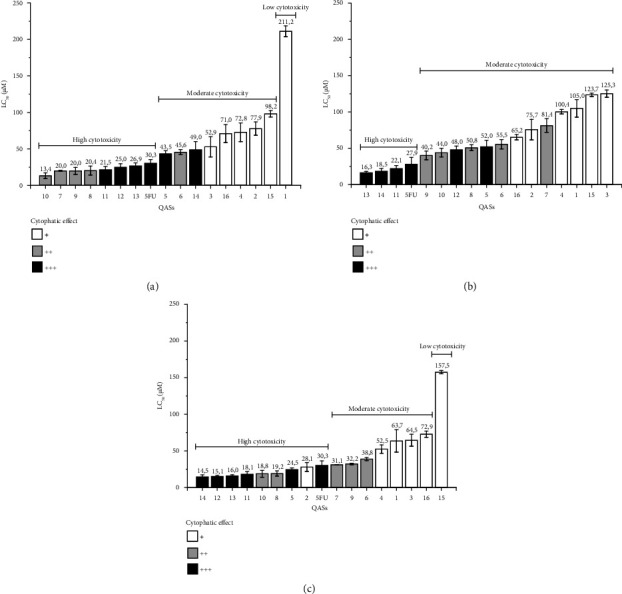
Cytotoxicity and cytopathic effect relationship of FQASs and QASs. The figure shows the cytotoxicity level of FQASs and QASs according to the median cytotoxic concentration (CC_50_) and the cytopathic effect in (a) SW948 Dukes' Type C, Grade III premetastatic colorectal cancer line; (b) SW48 Dukes' Type C, Grade IV in situ colorectal cancer line; (c) SW620 Dukes' Type C, Grade IV metastatic colorectal line. Cytopathic effect: mild (+), moderate (++), strong (+++).

FQASs **5–14** displayed significantly stronger cytotoxicity across CRC cell lines, with CC_50_ values ranging from 14.5 to 49 *μ*M and strong CPE (+++), indicating a pronounced reduction in cell viability (60%–90%). Notably, compound **5** had CC_50_ values of 43.5 *μ*M in SW48, 52.0 *μ*M in SW620, and 24.5 *μ*M in SW948, all with strong cytotoxic effects (+++). The reference compound, 5-FU, showed comparable cytotoxicity with CC_50_ values of 30.3 *μ*M in SW48, 27.9 *μ*M in SW620, and 30.3 *μ*M in SW948, along with strong CPE (+++). In contrast, the FQASs and QASs had a significantly reduced cytotoxic effect on MCRT and PBMC cell lines, where CC_50_ values were substantially higher, indicating lower toxicity. For example, compound **5** showed a CC_50_ of 121 *μ*M in MCRT and 34.8 *μ*M in PBMC. This trend was consistent across most compounds, including the reference 5-FU, which had a CC_50_ value of 944.7 *μ*M in MCRT and 38.7 *μ*M in PBMC (Figure 2).

The cytotoxicity of FQASs over the premetastatic CRC cell line SW948 (Dukes' Type C, Grade III) was moderate to high (Figure 2(a)). CC_50_ values for FQASs ranged between 211.2 *μ*M (compound **1**) and 13.4 *μ*M (compound **10**). Compounds **7–13** showed a cluster of similar CC_50_ values, exhibiting a slightly higher cytotoxicity than the reference drug 5-FU, which had a CC_50_ of 30.3 *μ*M. A notable observation was that compounds with strong cytotoxicity also exhibited a comparable CPE to 5-FU. For example, compounds 7–14 were marked as (+++) for strong cytotoxicity, suggesting a potential shared mechanism of action with 5-FU. Interestingly, the premetastatic CRC cell line SW948 appeared to be more susceptible to QASs **15** and **16**, which displayed moderate CC_50_ values. This suggests that QASs may have different mechanisms of action when compared to FQASs.

For the metastatic in situ CRC cell line SW48 (Dukes' Type C, Grade IV), FQASs demonstrated a wide range of cytotoxic effects, with CC_50_ values between 125.3 *μ*M (compound **3**) and 16.3 *μ*M (compound **13**) (Figure 2(b)). FQASs **11**, **13**, and **14** showed stronger cytotoxic effects than 5-FU (which had a CC_50_ of 27.9 *μ*M) with respective CC_50_ values of 22.1, 18.5, and 16.3 *μ*M. These three compounds also demonstrated strong CPEs (+++). Additionally, compounds **5** and **12** were also labeled as (+++) for strong CPE, correlating with their high cytotoxicity. In contrast, QAS **15** and **16** demonstrated only moderate cytotoxicity for SW48 cells.

The data revealed that FQASs showed a range of cytotoxic effects on the metastatic CRC cell line SW620 (Dukes' Type C, Grade IV) (Figure 2(c)). For FQASs, CC_50_ values spanned from 63.7 to 14.5 *μ*M, with compounds **13** and **14** again standing out for their low CC_50_ values, indicating higher cytotoxicity. The strong CPE (+++) observed in the more cytotoxic FQASs further reinforces the relationship between cytotoxicity and cytopathic damage. QASs **15** and **16**, while still effective, were less potent compared to FQASs, exhibiting higher CC_**50**_ values and only mild to moderate CPEs. Table 3 (second column) and Figure 2(b) also show that FQASs have moderate to highly cytotoxic effects for the metastatic in situ CRC cell line SW48, with CC_50_ values ranging from 125.3 to 16.3 mM. FQASs **11**, **13**, and **14** were slightly more cytotoxic than the reference 5-FU (CC_50_ values of 27.9 mM for 5-FU, 22.1, 18.5, and 16.3 mM for compounds **11**, **14**, and **13**, respectively). Again, the top more cytotoxic FQASs (**11**, **13**, and **14)** had a robust CPE and FQASs **5** and **12** (labeled as +++). QAS **15** and **16** had moderate cytotoxicity in these CRC cells.

Structurally, the most cytotoxic FQASs for SW948 cells share certain features: (i) a random distribution of cytotoxicity attributes for the C3-tethered and C4-tethered salts, with similar CC_50_ values; (ii) the presence of a trifluoromethyl substituent attached to either the para-position or meta-position of the aromatic rings, although six out of seven FQASs are para-substituted; (iii) the presence or absence of a halogen atom attached to one of the methyl groups of the quaternary ammonium head, with a random distribution of cytotoxicity patterns for chlorinated, iodinated, or nonhalogenated salts. FQAS **1** shows low cytotoxicity (CC_50_ value of 211.2 *μ*M), followed by **2**, which are (i) salts with three methylenes, (ii) with or without C-halogenate in the quaternary head, and (iii) nonfluorinated or monofluorinated in the aromatic rings. In agreement with this observation, for premetastatic SW948 cells, the carbon bond length is not highly relevant. Moreover, for the SW948 line, the presence or absence of a halogen atom attached to one of the quaternary head's methyl groups does not seem relevant, as cytotoxicity results from a complex combination of structural variables in the organic cation.

In the case of SW48 CRC cells, the most cytotoxic FQASs share certain features: (i) a four-methyl bond between the quaternary nitrogen atom and the vinyl moiety; (ii) a trifluoromethyl substituent on the aromatic rings, with para-substituted molecules slightly more cytotoxic than meta-substituted ones; (iii) the presence or absence of an iodine atom attached to one of the methyl groups of the quaternary ammonium head (CH_2_I vs. CH_3_).

A random distribution of cytotoxic behavior of the salts studied suggests that for this type of CRC cell line, the length of the bound carbon is not highly relevant. However, qualitatively, the salts bound to four methyls tend to be the most cytotoxic [38, 39], in addition to the experimental results shown in Table 3. The presence or absence of a halogen atom bound to one of the methyl groups of the quaternary head is not significantly relevant for cytotoxicity, suggesting that cytotoxicity results from a complex combination of structural variables in the organic cation. As mentioned, the cytotoxic FQASs **5** and **11**–**14** showed strong (+++) CPE. Both salts carry a chlorine atom (C-Cl pattern) on the quaternary ammonium head and an aromatic CF_3_ substituent (*meta* and *para*, respectively). It is important to note that the epigenetic characteristics of SW48 cells mean that they do not respond to a defined pathway to 5-FU [40] and would be expected to behave similarly in the presence of FQASs **11**, **13**, and **14**, despite being the most cytotoxic 5-FU [40]. Additionally, although the structure of 5-FU is dissimilar to that of FQASs, it is known to be an effective alternative for treating CRC and other cancers. As observed in our analyses, the behavior of these fluorinated salts is like that of 5-FU, suggesting that these molecules are optimal for developing an antitumoral activity. In practice, CRC patients with mutated *KRAS* and positive methylation status (epigenetic profile) do not benefit from 5-FU monotherapy because 5-FU is ineffective in CRC cells with this genotype, as in the SW48 cell line (Table 3).

Similarly, for the SW948 line, the cytotoxic FQASs share certain features: (i) a random distribution of cytotoxicity patterns for the length of the tether, but the four top-cytotoxic FQASs **11**–**14** are four-methylene tethered ones; (ii) the presence of fluorine or a CF_3_ substituent attached to the aromatic rings, either to the para- or meta-position, although five out of eight FQASs are trifluoromethyl-substituted salts, mainly in the para-position; (iii) whether or not a halogen atom, attached to one of the methyl groups of the quaternary ammonium head, with a random distribution of cytotoxicity patterns for chlorinated, iodinated, or nonhalogenated salts, suggesting that this halogen atom is not significantly relevant for cytotoxicity against SW620 CRC cells. Although there is no clear profile, it is strongly suggested that metastatic CRC cells respond more to (both) four-methylene tethered and para-CF_3_-substituted FQASs.

MCRT and PBMCs control cell lines have differential susceptibilities to FQASs and QASs compounds compared to 5-FU susceptibility profiles (Table 3, fifth and sixth columns). FQASs were reported to be 50%–90% more cytotoxic than 5-FU against TCRM (CC_50_ for 5-FU 944.7 mM; meanwhile, FQASs CC_50_ ranges from 419.4 mM to 93.5 mM). However, PBMC cells showed a susceptibility profile in the same order of magnitude between most FQASs and the reference compound 5-FU (CC_50_ 38.7 mM for 5-FU vs. CC_50_ ranging from 29.3 to 46.3 mM for FQASs). The observations regarding the QAS's cytotoxic effects against PBMC cells are comparable to the ones observed for FQASs in the same cell line, where QASs showed CC_50_ values comparable to the ones observed for 5-FU (CC_50_ values of 51.4, 53.9, and 93.2 mM for FQASs **10**, **13**, and **14**, respectively). Both QASs **15** and **16** were highly cytotoxic for PBMCs; meanwhile, the MCRT FQASs **15** had moderate cytotoxicity, while compound 16 was noncytotoxic.

When comparing the CC_50_ values obtained for nontumoral MCRT and the CC_50_ obtained for CRC, it can be noticed that the values are much lower for CRC cells than MCRT, which means that the toxicity is higher in CRC cells (Table 3). This selectivity in cytotoxicity for some cells or others is reflected in IS higher than one, which is calculated by dividing the CC_50_ value obtained in nontumoral cells (MCRT or PBMC) by the CC_50_ value obtained for each CRC cell.

All the FQASs exhibited more cytotoxicity than 5-FU against TCRM mice, with a random distribution of cytotoxicity patterns compared to the structural features approached. Although this is not a standout result, it is noteworthy that several FQASs exhibit lower cytotoxicity against healthy PBMC cells. Ten FQASs **3**, **4**, **6**, **7**, **8**, **10**, and **12**–**14** were less cytotoxic (or comparable) than 5-FU against healthy PBMC cells (Table 3). While FQASs **13** and **14** tend to be cytotoxic molecules against the three CRC cells SW48, SW948, and SW620, these salts resulted in the least cytotoxic for healthy control PBMC cells, which means that at least eight salts (**7**–**14**) exhibit better IS than 5-FU (Table 4). Furthermore, a fundamental level of selectivity was observed for all the FQASs when comparing their cytotoxicity on healthy TCRM cells versus cytotoxicity on the three CRC cells studied. Indeed, according to Table 3, FQASs **13** and **14** (mainly the latter) exhibited IS values comparable to 5-FU (fourth, fifth, and sixth columns, entries 13 and 14). The best IS value was found for FQASs **14**, which equals IS of 5-FU within experimental error (about the same cell lines). Although all FQASs and QASs also showed high cytotoxicity to PBMCs, the concentrations at which the mean toxic concentration is reached are higher than that observed for CRC cell lines. The cytotoxicity FQASs could be associated with mitochondrial damage because the MTT detects the activity of the mitochondrial enzyme succinate dehydrogenase; if this enzyme is altered, it is most likely that the mitochondria fail and generate cell death signals too early; nonetheless, this damage could be corrected, and cell recovery would occur. On the other hand, because these compounds are highly toxic, it is most likely that the damage is more significant than the repair events in the mitochondria. There is most likely no cell death due exclusively to mitochondrial damage but with additional adverse effects such as deterioration in other cellular organelles such as the membrane plasmatic [41].

The selectivity of FQAS and QAS for tumor cells over nontumor cells was calculated by comparing CRC cell lines to nontumor PBMC and MCRT cells. The calculated indexes of selectivity (IS) are summarized in Table 4. Notably, FQAS 13 exhibited significant selectivity for CRC cells, being up to 10 times more cytotoxic for SW948 cells and 18 times more cytotoxic for SW48 and SW620 cells. Similarly, FQAS 14 demonstrated high selectivity, with SI values of 22.7 for SW48 cells and 28.9 for SW620 cells, which correspond to noninvasive and invasive metastatic colorectal adenocarcinoma, respectively.

The majority of FQASs' selectivity index greater than 2 indicates that these compounds are more toxic to tumor cells than to nontumor cells, suggesting that they may be a good candidate in cancer therapy by minimizing damage to healthy cells.

As expected, 5-FU's antitumoral activity was selective for CRC cells instead of healthy MCRT, with IS values ranging between 31.2 and 33.9 (Table 4). Interestingly, no differences were observed in the compounds' selectivity for CRC cells and PBMCs, with IS values between 1.3 and 1.4.

The selectivity of FQASs **1**–**14** and QASs **15** and **16** between premetastatic (SW948), in situ metastatic SW48 cells, and invasive metastatic SW620 cells was also calculated by dividing the CC_50_ value obtained in noninvasive metastatic SW948 cells by the CC_50_ value obtained for in situ metastatic SW48 or invasive metastatic SW620 cells and, between in situ metastatic SW48 and invasive metastatic SW620 cells. The IS obtained are summarized in Table 5.

The IS values of most FQASs were < 1.0. Only FQASs **14**, **1**, and **13** had IS values of 2.6, 2.0, and 1.7, respectively (Table 5). Similarly, the comparison of the selectivity of FQASs and QASs between premetastatic versus invasive CRC cells showed that FQASs **14**, **1**, **2**, **5**, **13**, **12**, **7**, **4**, and **11** were selective for premetastatic cells with IS values of 3.4, 3.3, 2.8, 1.8, 1.7, 1.7, 1.6, 1.4, and 1.2, respectively. As expected, compound 5-FU showed no selectivity between CRC cells with values close to 1.0 (Table 5).

### 2.4. In Silico Analysis of Physicochemical Properties and Pharmacology of FQASs **1**–**14** and QASs **15** and **16**

Simplified Molecular Input Line Entry Specification formulas (SMILES) were generated for each FQAS and QAS and subsequently loaded to the ADMETLab2.0 platform to calculate the physicochemical and pharmacodynamic parameters and distribution. Calculated parameters are summarized in Table 6. The molar mass of FQASs and QASs ranged from 443.30 to 683.30 g/mol, with larger molecular weights being a common feature across the compounds. All FQASs had 1 hydrogen bond acceptor, while the number of hydrogen bond donors was zero for all compounds, indicating limited potential for hydrogen bonding with biological targets. The number of bond rotations varied slightly: 9 bond rotations for compound **5** and 10 bond rotations for compounds **11** and **13**. The variability in bond rotations may affect the molecular flexibility and interaction with biological systems.

The calculated polar topological surface area (**TPSA**) for all compounds was **0.000** Å^2^, indicating that these molecules have no polar surface area, which may hinder interactions with polar solvents and reduce the likelihood of crossing biological barriers like the blood–brain barrier (BBB). The LogS values, which reflect the aqueous solubility of the compounds, ranged from −4.373 to 0.033, indicating that all FQASs and QASs are poorly soluble in polar solvents. The LogP values, representing the partition coefficient between octanol and water (log octanol/water), indicate that FQASs and QASs are highly apolar molecules. Their high solubility in organic solvents suggests poor solubility in aqueous environments. The values for LogD and LogP at pH 7.4 were above optimal levels, ranging from 3.003 to 4.385, suggesting that these compounds are more lipophilic than recommended for drug-like properties.

The pharmacological properties of FQASs **2-16** were evaluated based on Lipinski's Rule of Five and Veber's criteria. All compounds were rejected due to their molar mass exceeding 400 g/mol and LogP values greater than 4, indicating they may have poor bioavailability and permeability. QASs **9**–**14** were predicted not to cross the BBB, which suggests limited utility in targeting central nervous system (CNS) disorders. High plasma protein binding (PPB) values (indicating strong binding to plasma proteins) were reported for most compounds, except for FQASs **1** and **2**, which had optimal PPB values < 90%, suggesting these two compounds may be better candidates for bioavailability.

The FQASs and QASs studied display high molecular weight (MW), poor aqueous solubility, and high lipophilicity, which leads to their rejection based on Lipinski's and Veber's rules for drug-likeness. While FQASs **9**–**14** cannot cross the BBB, they display high PPB values, whereas FQASs **1** and **2** have more favorable PPB properties. Despite their pharmacokinetic challenges, the lack of PAINS alerts suggests these compounds could be refined for specific, targeted applications.

Fluorine-containing compounds have emerged as a robust and practical tool in rational drug design, leveraging their advantages in potency and absorption, distribution, metabolism, excretion, and toxicity (ADMET) considerations [42]. Fluorine substitution is renowned for conferring exceptional electronic, physical, and biological attributes to organic molecules, making it a cornerstone in modern pharmaceuticals. This substitution often elevates pharmacological properties, including metabolic stability, membrane permeability, and receptor binding affinity. Incorporating fluorine into QASs holds promise for enhancing the potency and bioavailability of these compounds. Notably, approximately half of the top-selling drugs, or blockbuster drugs, incorporate fluorine-containing compounds. As a result, approximately half of the top-selling drugs, also known as blockbuster drugs, incorporate fluorine-containing compounds.

The in silico analyses provide information on physicochemical parameters, allowing an approach to some properties related to the potential pharmacology of the FQASs under study. Firstly, it was observed that all the FQASs evaluated are highly lipophilic and poorly soluble in polar solvents since, according to the maximum levels of bioavailability, they are out of the standard parameters that a drug should have to be administered orally. This observation is congruent with their chemical structure. Despite being QASs, their tail contains a long carbon chain and two aromatic rings, which make them more lipophilic. Furthermore, the trifluoromethyl (*p*-CF_3_ or *m*-CF_3_) or fluorine (*m*-F or *p*-F) groups located on both aromatic rings at the end of the chain are not sufficient to decrease these liposolubility values. It was observed that the MWs of these compounds range from 330 to almost 556.09 g/mol, indicating that they are heavy molecules compared to most of the compounds used orally. The TPSA value of 0.000 Å^2^ suggests minimal polar surface area, while the LogS values of −4.373 and 0.033 indicate poor aqueous solubility. These findings support the conclusion that FQASs are poorly soluble molecules in polar-type solvents. The LogP partition coefficient indicates that FQASs are highly apolar molecules with higher solubility in organic solvents. These align with the observed poor aqueous solubility and support the conclusion that FQASs are more soluble in nonpolar solvents. The values of LogD and LogP above the optimum for this parameter (ranging from 3.003 to 4.385) suggest that FQASs may have challenges in terms of distribution and permeability. The rejection of FQASs **2**–**16** based on their molar mass (> 400) and LogP (> 4) aligns with the Lipinski and Veber rules, which are commonly used guidelines for assessing the drug-likeness of compounds. FQASs **9**–**14** are not crossing the BBB but have high PPB values, which indicates potential challenges in terms of CNS penetration and distribution in the bloodstream. Lastly, the absence of alerts for PAINS suggests that FQASs do not exhibit problematic chemical interactions with various biological targets, which is a positive finding for their potential as drug candidates. These properties have proven to be a valid descriptor in many models and rules to quickly estimate absorption, distribution, metabolism, and excretion (ADME) properties, especially for crossing biological barriers such as absorption and accessing the brain or BBB [43].

The in vitro experiments provide a controlled environment to evaluate the efficacy and toxicity profiles of FQAS candidates, laying the foundation for selecting lead compounds for further preclinical and in vivo assessments. Moreover, in vitro assays enable screening a broad range of FQAS derivatives to identify potent candidates with promising antitumor properties, enhancing the efficiency of subsequent in vivo studies. Additionally, in vitro studies contribute to establishing structure–activity relationships and identifying lead compounds with favorable pharmacokinetic and pharmacodynamic profiles for subsequent in vivo efficacy and safety evaluations. Therefore, in vitro cytotoxicity and pharmacology data of FQAS against CRC cell lines presented in this work are promising.

Nonetheless, it is crucial to recognize that the study lacks comprehensive in vivo validation that would complement the existing in vitro findings and provide a more holistic understanding of the therapeutic potential of FQAS. The current study's limitation of in vivo data is mainly due to the initial approach: identifying lead molecules among a battery of compounds to preselect the most promising ones. This limitation will be addressed in future studies, where priority will be given to in vivo experiments to bridge the gap between preclinical findings and potential clinical applications of FQAS in treating CRC. The absence of comprehensive in vivo data currently limits the extrapolation of our findings to clinical settings, underscoring the preliminary nature of this study. Addressing this limitation through future research initiatives will strengthen the scientific basis of our work and provide a more complete understanding of the therapeutic mechanisms of FQAS.

## 3. Conclusions

The synthesized FQASs **9**–**14**, particularly those containing CF_3_, show promising potential as antitumor agents for advanced stages of CRC, such as Dukes' Type C and Grade III. The selectivity of FQASs toward CRC tumor cells, without affecting PBMC, makes them attractive candidates for further development. Even though 5-FU is a widely used and effective drug for treating CRC, its long-term efficacy diminishes due to several limitations, mainly associated to drug resistance, variable patient response, and side effects. These limitations highlight the need for new compounds to overcome drug resistance, reduce toxicity, and improve treatment outcomes for CRC patients.

The FQASs **9**–**14** containing CF_3_ represent potential candidates for antitumor agents against CRC, particularly in advanced stages like Dukes' Type C and Grade III. The observed selectivity of FQASs toward tumor cells over normal tissue, without affecting PBMC, suggests the need for strategies facilitating direct tissue delivery while circumventing systemic circulation. Addressing potential challenges associated with utilizing FQASs as drug candidates may involve employing specific release formulations, such as enterically coated tablets or capsules resistant to gastric juices. Additionally, utilizing prodrugs activated by colonic enzymes or administering drugs directly into the colon via techniques like enemas could enhance efficacy. Targeted delivery systems, such as nanoparticles or functionalized microspheres, tailored to release the drug selectively in the colon while minimizing systemic absorption, present further avenues for exploration.

The synthesized FQASs demonstrate promising cytotoxicity and selectivity against CRC cells, positioning them as potential candidates for therapeutic development. The observed correlations between genotype and cellular phenotype suggest that FQASs could be tailored to create personalized treatments for specific subgroups of CRC patients. However, further studies are needed to optimize the structure of FQASs to enhance their pharmacological activity, improve their anticancer efficacy, and minimize side effects linked to chemical groups not involved in antitumor mechanisms. Additionally, advancing preclinical studies using relevant CRC models is crucial to comprehensively assess the efficacy and safety of FQASs in CRC treatment. Moreover, metabolomic research should focus on exploring the metabolic pathways modulated by FQASs in CRC cell lines (SW620, SW48, and SW948). This will provide deeper insights into the mechanisms of action and the accessory pathways contributing to the antitumor response of these compounds. Such studies hold promise for developing new treatment modalities, especially for CRC patients resistant to current therapies. The ability to tailor treatments based on individual cancer characteristics highlights the growing importance of precision medicine in improving patient outcomes.

## 4. Experimental

### 4.1. Chemistry

#### 4.1.1. General Procedures for the Synthesis of FQASs and QASs

The QASs and FQASs were synthesized and prepared according to the well-known four-step synthetic sequence, which involves (a) in situ generation of trifluoromethyl- or fluorine-containing aryl magnesium Grignard reagents (either meta- or para-substituted) and their reaction with ethyl *ω*-bromoesters; (b) acid-catalyzed dehydration of the resulting tertiary *α*,*α*-bis-aryl-*ω*-bromoalcohols, giving rise to the respective *ω*-bromoolefins; (c) classical S_N_2 displacement of the bromide group by aqueous dimethylamine, to generate the respective tertiary amines; and (d) finally, reaction with either CH_2_I_2_ or ICH_2_Cl or CH_3_I in acetonitrile to produce iodide salts with -CH_2_I, -CH_2_Cl, or -CH_3_ groups respectively bound to the quaternary nitrogen [22, 38, 45].

The salts were crystallized from isopropanol–water binary mixtures and characterized by MS, ^1^H, and ^13^C NMR. Crystallographic analysis of representative QA's salts was performed, and signals are in accordance with our previous results reported elsewhere [22, 45].

The compounds were solubilized in dimethyl sulfoxide (DMSO, Fischer Chemical, NJ, USA) at 50,000 *μ*g/mL and stored at −20°C until use. From the stock solution, six serial 1:2 double dilutions of each compound were prepared to start with concentrations of 100, 50, 25, 12.5, 6.25, and 3.125 *μ*g/mL, respectively. 5-FU, one of the conventional drugs to treat CRC [34], was used as an antitumor compound control at concentrations ranging between 800 and 0.625 *μ*M.

### 4.2. Pharmacological/Biological Assays

#### 4.2.1. Cell Lines Phenotype and Culture Conditions

SW948 (Dukes' Type C, Grade III, premetastatic colorectal adenocarcinoma—CCL-237), SW48 (Dukes' Type C, Grade IV, noninvasive colorectal adenocarcinoma—CCL231), and SW620 (Dukes' Type C, Grade IV invasive colorectal adenocarcinoma—CCL227) (American Type Culture Collection Manassas, VA, USA) were maintained in standard conditions at 37°C, without a CO_2_ atmosphere. PBMCs and MCRT were used as nontumoral control cells. PBMCs were obtained by healthy donors, separated using Histopaque-1077 (Sigma-Aldrich) and cultured in RPMI-1640 medium (Sigma-Aldrich) supplemented with 10% SFB and 1% penicillin/streptomycin [46]. In turn, MCRT were obtained from healthy BALB/c mice; for this, mice were euthanized with CO_2_ after previous sedation, the colorectal tissue removed, cleaned up, and cut into 2 × 2 mm pieces, suspended in supplemented DMEM, and used immediately after collection. Both PBMC and MCRT were used as nontumoral control cells to assess the selective toxicity of FQASs. Moreover, the inclusion of PBMC also served to assess the systemic toxicity of FQAS.

#### 4.2.2. Analysis of Genetic and Epigenetic Profiling of CRC Cell Lines

The genetic features of the CRC cell lines were analyzed using the information available in the ATCC database (https://www.atcc.org/products/tcp-1007) and the Taiwanese Igrcid cell bank (https://igrcid.ibms.sinica.edu.tw/cgi-bin/cell_line_view.cgi). The epigenetic profile in these cell lines was verified according to parameters related to MSS or MSI, CIN, and CIMP reported in the scientific literature [39, 47–49] and the Cellosaurus database (https://www.expasy.org).

#### 4.2.3. In Vitro and Ex Vivo Cytotoxicity of FQASs and QASs

The cytotoxic activity of synthesized FQASs and QASs was studied by measuring cell viability/mortality, determined with the MTT (3-(4,5-dimethylthiazol-2-yl)-2,5-diphenyl tetrazole bromide) method (VWR Chemicals and Reagents Avantor), upon exposure of each cell line to six serial dilutions of the different compounds independently [50]. Unexposed cells were included as viability controls, and cells exposed to 5-FU were included as cytotoxicity controls. Briefly, cells were adjusted to 200,000 cells/mL in the corresponding supplemented culture medium, and 100 *μ*L (20,000 cells/well) were dispensed into each 96-well cell culture plate (Nunc, Thermo Fisher Scientific). Twelve hours after seeding the monolayer, 100 *μ*L dilution of each compound was added to each well; the plates containing CRC were incubated at 37°C in the absence of CO_2_, while plates with PBMCs and MCRT were incubated at 37°C and 5% CO_2_ atmosphere. For MCRT, each piece was placed into each well of a 96-well plate and exposed to each dilution of the FQASs and QASs. After 48 h of incubation, 30 *μ*L/well of MTT (5 mg/mL) was added, and plates were left at 37°C for 3 h, protected from light. After this time, 100 *μ*L/well of DMSO was added to solubilize the formazan crystals formed. Plates were read at 570 nm on a Varioskan plate reader (Thermo Fisher), and nonspecific absorbance was corrected by subtracting the optical densities (OD) of the blank solutions corresponding to the supplemented culture media (RPMI 1640, DMEM, or L-15 media) and 0.5% DMSO. Each concentration of the FQASs, QASs, and controls was evaluated in duplicate in three independent experiments.

The percentages of viability (equation (1)) and mortality (equation (2)) were calculated with the net OD:

(1) $$\begin{array}{c} \%\;v\text{iability}=\left(\text{OD treated cell}÷\text{nontreated cells}\right)\times 100, \end{array}$$

(2) $$\begin{array}{c} \%\;m\text{ortality}=100-\%\;v\text{iability}. \end{array}$$

The %mortality data for each compound concentration was recorded, and the median cytotoxic concentration (CC_50_), corresponding to the concentration at which 50% of cell death occurs, was calculated using GraphPad Prism 8 software (GraphPad, Software, Inc., La Jolla, CA, USA). The cytotoxic activity of the evaluated FQASs and QASs was classified as high, moderate, or low according to the CC_50_ values obtained for compound 5-FU as follows: high cytotoxicity for CC_50_ values < 50 *μ*M, moderate for CC_50_ values > 50 and < 150 *μ*M, and low for values > 150 *μ*M. Lastly, the index of selectivity (IS) was calculated by dividing the CC_50_ value obtained for control cells (MCRT and PBMCs) by the CC_50_ value obtained for each CRC cell phenotype using the formula: IS = CC_50_ (MCRT or PBMC) ÷ CC_50_ (SW948, SW48 or SW620).

#### 4.2.4. In Silico Analysis of Physicochemical Properties and Pharmacology

The open-access multiparameter platform ADMETLab2.0 (https://admetmesh.scbdd.com) [51] was utilized to analyze the physicochemical and distribution parameters of FQASs and QASs, as well as their pharmacological properties according to the Lipinski et al. [52] and Veber et al. [53] rules. The physicochemical properties analysis included parameters such as MW, number of hydrogen donors (nHD), number of hydrogen acceptors (nHA), number of rotations (nRot), TPSA, logarithm of aqueous solubility (LogS), log octanol/water partition coefficient (LogP), and LogP at physiological pH 7.4 (LogD). Distribution parameters encompassed the assessment of BBB permeability and PPB. Lastly, the oral bioavailability of compounds was evaluated based on the compliance with Lipinski's rule of five (MW < 500, log *p* < 5, hydrogen bond donors < 5, and hydrogen bond acceptors < 10) [52] and Veber's rule (rotatable bonds [RTB] < 10 and TPSA < 140 Å^2^) [53].

#### 4.2.5. Statistical Analysis

All experimental assays were conducted in duplicate across a minimum of three independent experiments. For comparisons between treatment groups, one- or two-way ANOVA with Tukey's or Dunnett's post hoc test, involving multiple treatments, was performed after confirming the normal distribution of the data calculated using GraphPad Prism 8 software (GraphPad, Software, Inc., La Jolla, CA, USA). Statistical significance was determined with a *p* value < 0.05 (⁣^∗^), < 0.01 (⁣^∗∗^), and < 0.001 (⁣^∗∗∗^) considered as statistically significant.

## Figures and Tables

**Table 1:** Genetic alterations status of colorectal cancer cell lines SW620, SW48, and SW948.

Cell line	Mutant gene	Encoded protein	Protein function	Zygosity	Mutation	Amino acid change	Platform	
							ATCC	IGRhCellID
SW948	*KRAS*	K-Ras	Cell proliferation and differentiation	Heterozygous	182A > T	Q61L	x	x
	*PIK3CA*	p110 alpha (p110*α*) protein	Cell proliferation and migration	Heterozygous	1624G > A	E542K	x	x
	*APC*	APC protein	Tumor suppression	Heterozygous	3340C > T	R1114⁣^∗^	x	x
				Heterozygous	4285C > T	Q1429⁣^∗^	x	x
	*TP53*	Tumor protein 53	Tumor suppression	ND	412 stop at 122	Gly117FS	—	x

SW620	*KRAS*	K-Ras	Cell proliferation and differentiation	Homozygous	35G > T	G12V	—	x
				Heterozygous	182A > T	Q61L	—	x
	*TP53*	Tumor protein 53	Tumor suppression	Homozygous	818G > A	R273H	—	x
				Homozygous	925C > T	P309S	—	x
				ND	881G > A	Arg273His	—	x
				ND	988C > T	Pro309Ser	—	x
	*APC*	APC protein	Tumor suppression	Homozygous	4012C > T	Q1338	—	x
				Heterozygous	3340C > T	p.R1114^∗^	—	x
				Heterozygous	4285C > T	p.Q1429^∗^	—	x
	*SMAD4*	TGFB	Tumor suppression	Homozygous	5G > C	—	—	x
	*PIK3CA*	p110 alpha (p110*α*) protein	Cell proliferation and migration	Heterozygous	1624G > A	p.E542K	—	x

SW48	*CTNNB1*	*β*-Catenin	Cell proliferation and differentiation	Heterozygous	98C > A	S33Y	x	x
	*EGFR*	Epidermal growth factor receptor	Cell proliferation and cell survival	Heterozygous	2155G > A	G719S	x	x
	*FBXW7*	F-box and WD repeat domain-containing 7	F-box protein family	Heterozygous	2001delG	S668fs^∗^39	x	—
	*TP53*	Tumor protein 53	Tumor suppression	ND	805C > T	Arg248Trp	—	x

*Note:* Data extracted from The Cancer Genome Atlas (TCGA) [30] at the NIH database (https://www.portal.gdc.cancer.gov), the American Type Culture Collection—ATCC (https://www.atcc.org/), and the IGRhCellID (https://igrcid.ibms.sinica.edu.tw).

Abbreviations: *APC* gene, adenomatous polyposis coli; *CTNNB1* gene, catenin beta-1; *EGFR* gene, epidermal growth factor receptor; *FBXW7* gene, F-Box and WD Repeat Domain Containing 7; *KRAS* gene, Kirsten rat sarcoma viral oncogene; *PIK3CA* gene, phosphatidylinositol-4,5-bisphosphate 3-kinase catalytic subunit alpha; *SMAD4* gene, mothers against decapentaplegic member 4; *TP53* gene, tumoral protein p53.

**Table 2:** Epigenetic status of SW48, SW620, and SW948 cell lines^a^.

Cell line	Disease	Stage	MSI/MSS status	CIMP Panel 1	CIMP Panel 2	CIN
SW948	Adenocarcinoma	Premetastatic	MSS+	ND	ND	ND
SW48	Adenocarcinoma	Metastatic in situ	MSI+	+	+	−
SW620	Adenocarcinoma	Metastatic invasive	MSS+	+	+	−

*Note:* The microsatellite instability/stability status in CRC SW948, SW48, and SW620 cell lines. Panel 1 (*CDKN2A(p16)*, *MINT1*, *MINT2*, *MINT31*, and *MLH1*); Panel 2 (CACNA1G, IGF2, NEUROG1, RUNX3 and *SOCS1*).

Abbreviations: CIMP, CpG island methylator phenotype; CIN, chromosomal instability pathway; MSI, microsatellite instability; MSS, microsatellite stability; ND, no data available.

^a^Information was taken from the Cellosaurus platform (https://www.expasy.org) and [30].

**Table 3:** Cytotoxicity and cytopathic effect of FQASs and QASs in different cell lines.

Compound	CC_50_ in *μ*M (CPE)^a^				
	SW948	SW48	SW948	MCRT	PBMC
**1**	211.2 ± 7.4 (+)	105.0 ± 12.0 (+)	63.7 ± 15.3 (+)	212.0 ± 4.0 (NA)	29.8 ± 2.4 (NA)
**2**	77.9 ± 9.1 (+)	75.7 ± 14.2 (+)	28.1 ± 6,1 (+)	204.7 ± 14.5 (NA)	35.6 ± 6.6 (NA)
**3**	52.9 ± 13.8 (+)	125.3 ± 5.1 (+)	64.5 ± 8.1 (+)	129.7 ± 20.0 (NA)	39.7 ± 7.7 (NA)
**4**	72.8 ± 12.8 (+)	100.4 ± 3.0 (+)	52.5 ± 5.6 (+)	106.8 ± 4.5 (NA)	45.1 ± 7.3 (NA)
**5**	43.5 ± 4.0 (+++)	52.0 ± 9.2 (+++)	24.5 ± 2.3 (+++)	121.0 ± 5.4 (NA)	34.8 ± 6 (NA)
**6**	45.6 ± 3.6 (++)	55.5 ± 6.4 (++)	38.8 ± 2.1 (++)	129.0 ± 11.3 (NA)	44.8 ± 6.4 (NA)
**7**	20.0 ± 0.8 (++)	81.4 ± 9.4 (++)	31.1 ± 0.2 (++)	123.2 ± 9.2 (NA)	46.3 ± 7.5 (NA)
**8**	20.4 ± 6.2 (++)	50.8 ± 4.0 (++)	19.2 ± 3.5 (++)	102.0 ± 5.0 (NA)	42.3 ± 9.3 (NA)
**9**	20.0 ± 4.7 (++)	40.2 ± 6.0 (++)	32.2 ± 1.0 (++)	206.1 ± 19.4 (NA)	30.6 ± 2.1 (NA)
**10**	13.4 ± 3.9 (++)	44.0 ± 6.0 (++)	18.8 ± 4.7 (++)	119.4 ± 8.7 (NA)	51.4 ± 5.3 (NA)
**11**	21.5 ± 4.5 (+++)	22.1 ± 4.0 (+++)	18.1 ± 3.7 (+++)	93.5 ± 13.9 (NA)	33.4 ± 2.0 (NA)
**12**	25.0 ± 4.8 (+++)	48.0 ± 5.1 (+++)	15.1 ± 1.5 (+++)	110.6 ± 16.7 (NA)	40.5 ± 9.5 (NA)
**13**	26.9 ± 4.0 (+++)	16.3 ± 2.0 (+++)	16.0 ± 1.5 (+++)	> 292.7 (NA)	53.9 ± 3.0 (NA)
**14**	49.0 ± 11.1 (+++)	18.5 ± 3.6 (+++)	14.5 ± 2.9 (+++)	419.4 ± 15.0 (NA)	93.2 ± 13.1 (NA)
**15**	98.2 ± 4.3 (+)	123.7 ± 2.3 (+)	157.5 ± 2.3 (+)	255.9 ± 14.0 (NA)	29.3 ± 1.4 (NA)
**16**	71.0 ± 12.4 (+)	65.2 ± 3.7 (+)	72.9 ± 4.2 (+)	134.9 ± 11.8 (NA)	38.8 ± 5.6 (NA)
**5-FU**	30.3 ± 5.2 (+++)	27.9 ± 9.6 (+++)	30.3 ± 6.0 (+++)	944.7 ± 43.7 (NA)	38.7 ± 7.0 (NA)

*Note:* Data represent the mean ± SD of three independent trials with two technical replicates of the CC_50_ values that provide a quantitative measure of cytotoxicity, with lower values indicating higher toxicity. The CPE describes the qualitative effects on cell viability, with categories ranging from mild to strong cytotoxic effects of the median cytotoxic concentration (CC_50_), where the higher the CC_50_ value is, the less cytotoxic the compound is. SW620: Dukes' type C, invasive metastatic colorectal adenocarcinoma; SW48: Dukes' type C, grade IV noninvasive (in situ) metastatic colorectal adenocarcinoma; SW948: Dukes' type C, grade III, pre-metastatic colorectal adenocarcinoma; +: Mild effect (10–30% reduction in viability); ++: Moderate effect (30–60% reduction in viability); +++: Strong effect (60–90% reduction in viability); (NA): not applicable in non-adherent MCRT and PBMC.

Abbreviations: MCRT, healthy murine colorectal tissue; PBMC, peripheral blood mononuclear cells.

^a^CPE, cytopathic effect.

**Table 4:** Selectivity of FQASs and QASs for CRC cells vs. nontumoral cells MCRT and PBMC.

Compound	Index of selectivity					
	MCRT			PBMC		
	SW948	SW48	SW620	SW948	SW48	SW620
**1**	1.0	2.0	3.3	0.1	0.3	0.5
**2**	2.6	2.7	7.3	0.5	0.47	1.3
**3**	2.5	1.0	2.0	0.8	0.32	0.6
**4**	1.5	1.1	2.0	0.6	0.45	0.9
**5**	2.8	2.3	4.9	0.8	0.66	1.4
**6**	2.8	2.3	3.3	1.0	0.80	1.2
**7**	6.2	1.5	4.0	2.3	0.57	1.5
**8**	5.0	2.0	5.3	2.1	0.8	2.2
**9**	10.3	5.1	6.4	1.5	0.8	1.0
**10**	8.9	2.7	6.4	3.8	1.2	2.7
**11**	4.4	4.2	5.2	1.6	1.5	1.9
**12**	4.4	2.3	7.3	1.6	0.8	2.7
**13**	> 10.9	> 18.0	> 18.3	2.0	3.3	3.4
**14**	8.6	22.7	28.9	1.9	5.0	6.4
**15**	2.6	2.1	1.6	0.3	0.2	0.2
**16**	1.9	2.1	1.9	0.6	0.6	0.5
**5-FU**	31.2	33.9	31.2	1.3	1.4	1.3

*Note:* Data correspond to the ratio between the CC_50_ for cells with the normal phenotype (MCRT and PBMC) and the CC_50_ for CRC cells (SW948, SW48, and SW620). SW948: Dukes' type C, grade III, pre-metastatic colorectal adenocarcinoma; SW48: Dukes' type C, grade IV noninvasive metastatic colorectal adenocarcinoma; SW620: Dukes' type C, invasive metastatic colorectal adenocarcinoma. IS = CC_50_ (MCRT or PBMC) ÷ CC_50_ (SW948, SW48 or SW620).

Abbreviations: 5-FU, 5-fluorouracil; MCRT, healthy murine colorectal tissue; PBMC, peripheral blood mononuclear cells.

**Table 5:** Selectivity of FQASs and QASs for premetastatic SW948 CRC cells vs. in situ metastatic SW48 and invasive metastatic SW620 CRC cells.

Compound	Index of selectivity		
	SW948 vs. SW48a	SW948 vs. SW620b	SW48 vs. SW620c
**1**	2.0	3.3	1.7
**2**	1.0	2.8	2.7
**3**	0.4	0.8	1.9
**4**	0.7	1.4	1.9
**5**	0.8	1.8	2.1
**6**	0.8	0.8	1.4
**7**	0.2	1.6	2.6
**8**	0.4	1.1	2.6
**9**	0.5	0.6	1.2
**10**	0.3	0.7	2.3
**11**	1.0	1.2	1.2
**12**	0.5	1.7	3.2
**13**	1.7	1.7	1.0
**14**	2.6	3.4	1.3
**15**	0.8	0.6	0.8
**16**	1.1	1.0	0.9
**5-FU**	1.1	1.0	0.9

*Note:* Data correspond to the ratio between the CC_50_ for CRC cells SW948 and SW48, SW948 and SW620, and SW48 and SW620. SW948: Dukes' type C, grade III, pre-metastatic colorectal adenocarcinoma; SW48: Dukes' type C, grade IV noninvasive metastatic colorectal adenocarcinoma; SW620: Dukes' type C, invasive metastatic colorectal adenocarcinoma.

Abbreviation: 5-FU, 5-fluorouracil.

^a^IS = CC_50_ SW948 ÷ CC50 SW48.

^b^IS = CC_50_ SW948 ÷ CC_50_ SW620.

^c^IS = CC_50_ SW48 ÷ CC_50_ SW620.

**Table 6:** Physicochemical parameters and medicinal chemistry parameters in silico analysis of the FQAS and QAS.

Compound	Physicochemical parameters								Medicinal chemistry				
	MW	nHA	nHD	nRot	TPSA (Å^2^)	LogS	LogP	LogD	Lipinsky rule	Veber rule	PAINS alert	BBB-penetrance	PPB %
**1**	457.3	1	0	7	0	−0.033	3.894	3.003	A	A	No	Yes	87.43
**2**	569.2	1	0	7	0	−0.596	3.847	3.217	R	R	No	Yes	87.32
**3**	491.8	1	0	8	0	−1.47	4.204	3.470	R	R	No	Yes	90.89
**4**	583.2	1	0	8	0	0.1.048	4.221	3.295	R	R	No	Yes	89.80
**5**	577.7	1	0	9	0	−3.711	4.870	4.307	R	R	No	No	95.72
**6**	669.2	1	0	9	0	−3.413	4.899	4.177	R	R	No	No	94.91
**7**	557.3	1	0	9	0	−2.618	4.931	4.141	R	R	No	No	5.32
**8**	577.8	1	0	9	0	−4.142	5.024	4.385	R	R	No	No	95.88
**9**	669.2	1	0	9	0	−3.965	5.049	4.242	R	R	No	No	95.07
**10**	557.3	1	0	9	0	−3.632	5.073	4.141	R	R	No	No	95.45
**11**	683.3	1	0	10	0	−3.827	5.277	4.156	R	R	No	No	95.45
**12**	591.8	1	0	10	0	−4.373	5.415	4.344	R	R	No	No	96.37
**13**	683.2	1	0	10	0	−4.221	5.424	4.220	R	R	No	No	95.63
**14**	571.3	1	0	10	0	−4.054	5.445	4.160	R	R	No	No	96.02
**15**	406,1	1	0	7	0	−0.027	3647	2935	A	R	No	Yes	85.23
**16**	420,1	1	0	8	0	−0.248	3982	2992	A	R	No	Yes	88.09
Accepted values	< 400	0–12	0–7	0–11	0–140	−4.0 to 0.5	0–3	1–3	—	—	No	No	< 90

*Note:* Lipinsky's and Veber's rules accepted values.

Abbreviations: A, accepted; BBB, blood–brain barrier; LogD, LogP at physiological pH 7,4; LogP, logarithm of the octanol/water partition coefficient; LogS, logarithm of the aqueous solubility; MW, molar weight; nHA, number of acceptor hydrogens; nHD, number of donor hydrogens; nRot, number of bonds; P, probably; PAINS, Pan-Assay INterference Substances; PPB, plasma protein binding; R, rejected; TSPA, topological polar surface area.

## Data Availability

The data that support the findings of this study are available from the corresponding author upon reasonable request.
